# Pollutants, including Organophosphorus and Organochloride Pesticides, May Increase the Risk of Cardiac Remodeling and Atrial Fibrillation: A Narrative Review

**DOI:** 10.3390/biomedicines11092427

**Published:** 2023-08-30

**Authors:** Ewen Le Quilliec, Alexia Fundere, Doa’a G. F. Al-U’datt, Roddy Hiram

**Affiliations:** 1 Department of Medicine, Faculty of Medicine, University of Montreal, Montreal, QC H3T 1J4, Canada; ewen.lequilliec@icm-mhi.org; 2 Research Center, Montreal Heart Institute, Montreal, QC H1T 1C8, Canada; fundere.alexia@gmail.com; 3 Department of Physiology and Biochemistry, Faculty of Medicine, Jordan University of Science and Technology, Irbid 22110, Jordan; dgaludatt@just.edu.jo

**Keywords:** atrial fibrillation, environmental pollution, pesticide, cardiotoxicity

## Abstract

**Highlights:**

**What are the main findings?**

- Abnormally increased concentrations of ingested or inhaled pollutants can lead to cardiac oxidative stress and inflammation.
- Untreated cardiac inflammation promotes myocardial fibrosis and cardiac arrhythmias.

**What is the implication of the main findings?**

- Patients hospitalized for acute pesticide poisoning often suffer from episodes of atrial or ventricular fibrillation.
- Management of pollutant poisoning associated with AF includes detoxification (i.e., gastric lavage) and prompt rhythm control.

**Abstract:**

Atrial fibrillation (AF) is the most common type of cardiac rhythm disorder. Recent clinical and experimental studies reveal that environmental pollutants, including organophosphorus–organochloride pesticides and air pollution, may contribute to the development of cardiac arrhythmias including AF. Here, we discussed the unifying cascade of events that may explain the role of pollutant exposure in the development of AF. Following ingestion and inhalation of pollution-promoting toxic compounds, damage-associated molecular pattern (DAMP) stimuli activate the inflammatory response and oxidative stress that may negatively affect the respiratory, cognitive, digestive, and cardiac systems. Although the detailed mechanisms underlying the association between pollutant exposure and the incidence of AF are not completely elucidated, some clinical reports and fundamental research data support the idea that pollutant poisoning can provoke perturbed ion channel function, myocardial electrical abnormalities, decreased action potential duration, slowed conduction, contractile dysfunction, cardiac fibrosis, and arrhythmias including AF.

## 1. Introduction

Atrial fibrillation (AF) is the most common and important type of cardiac arrhythmia [1]. Aging and conditions like diabetes, obesity, hypertension, lung disorders, myocardial infarction, or unhealthy lifestyle habits such as abuse of alcohol or smoking, have been designated among major AF risk factors [2,3,4,5,6,7,8]. AF is associated with severe complications such as stroke, heart failure, and sudden death [1]. AF patients are subjected to increased morbidity and mortality causing serious degradation of their quality of life [1,9].

Mounting evidence suggests that environmental factors, including exposure to air pollution, pesticides, herbicides, or passive smoking, are responsible for serious cardiotoxicity contributing to enhancing the risk of AF [10,11,12]. The mechanisms underlying the development of AF substrate following exposure to pollutants remain poorly described. It is suspected that air pollutants, pesticides, or herbicides could provoke myocardial remodeling leading to inflammation, cardiac fibrosis, and electrical changes that may participate in increasing the risk of cardiac arrhythmias and AF [13,14,15].

During the last two decades, significant advancements have helped to ameliorate the management of cardiac arrhythmias which contributed to improving AF patients’ quality of life [9]. However, more efforts need to be accomplished to efficiently prevent and cure cardiac arrhythmias and AF. Hence, a better understanding of the impact of environmental pollution on the incidence of AF may lead to the discovery of new preventive and curative therapeutic approaches.

This paper aims to report the recent knowledge about the mechanisms relating to environmental pollution and AF. In this review, we discussed the role of acute and chronic exposure to pollutants on the occurrence of AF. We explored clinical and experimental studies suggesting involvement of inflammation and cardiac fibrosis in the association between AF and pollutant exposure. We finally reported the current knowledge and potential future perspectives in the management of patients with AF caused by pollutant exposure.

## 2. Methods

We provide a comprehensive narrative synthesis of evidence extracted from the existing literature. This evidence comes from clinical and research reports purposefully identified as related to pollutant poisoning and cardiovascular disease, including cardiac rhythm disorder and AF. Papers references in this narrative review of literature were peer-reviewed and critically evaluated based on the methodological quality and consistency of results and conclusions. Eligible articles reviewed in the current article were all published previously and indexed in scientific databases until July 2023.

This narrative review comprises an introductory and contextualizing paragraph, followed by a discussion on the association between environmental pollutants and the incidence of cardiac disorders including atrial fibrillation. The following search formula: (“pollutant” OR “pesticide” OR “herbicide”) [title/abstract] + (“heart”, “cardiac”) [title/abstract] + (“atrial fibrillation”) was used to search in electronic databases such as Medline, PubMed, ScienceDirect, and Scopus. Articles with English full texts only were reviewed. Primarily, abstracts obtained from previously cited databases were analyzed to scrutinize studies relevant to environmental pollution, including air pollution, occupational toxic compounds, pesticides, or insecticides, in the context of cardiac diseases. We considered relevant articles in which pollutants (according to the definition of the World Health Organization [WHO]) were studied or applicable to heart and cardiac diseases. To describe, contextualize, and propose perspectives of research and practice, about important concepts or essential mechanisms relevant to the current theme of environmental pollution in cardiac arrhythmias, additional references have been included to strengthen the discussion and characterize potential biochemical cascades that may underly the pathophysiological mechanisms.

## 3. Evidence of the Association between Air Pollution and Cardiac Arrhythmias

### 3.1. Definition, Nomenclature, and Sources of Air Pollutants

**Figure 1:**
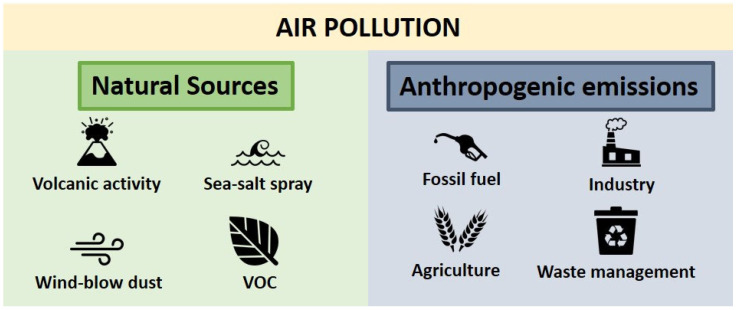
Main sources of air pollutants. In general, air pollution is generated by either natural or anthropogenic emissions or a mix of both.

Air pollution is defined as the acute or persistent presence of inhalable dangerous and potentially health-threatening substances in the atmosphere [16]. Air pollutants mainly originate from natural sources, anthropogenic emissions, or a mix of both [17]. Natural sources of air pollutants include volcanic activity, sea salt spray, windblown dust, or plants’ volatile organic compounds (VOC) [18,19,20]. Anthropogenic emissions are, by definition, provoked by human activity and include fossil-fuel burning, industrial processes, agriculture, or waste management [21,22,23] (Figure 1).

**Figure 2:**
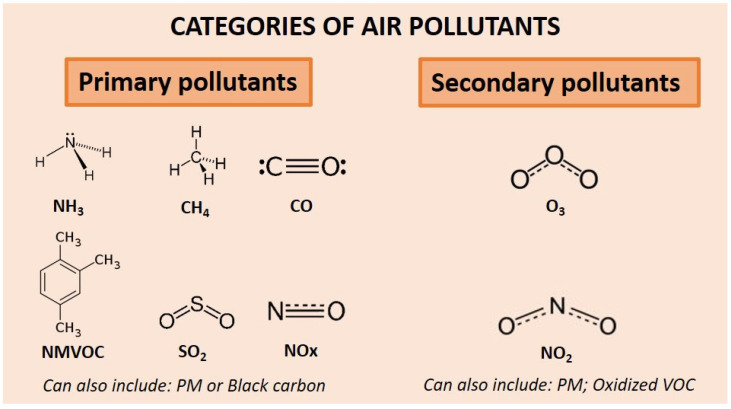
A list of primary and secondary pollutants. In general, air pollution is generated by either natural or anthropogenic emissions or a mix of both. CH_4_: Methane; CO: Carbon Monoxide; NH_3_: Ammoniac; NO_2_: NMVOC: Non-Methane Volatile Organic Compounds; Nitrogen Dioxide; NOx: Nitrogen Oxides; O_3_: Ozone; SO_2_: Sulfur Dioxide; VOC: Volatile Organic Compounds.

Air pollutants can be categorized into two groups: primary pollutants (emitted directly in the atmosphere) and secondary pollutants (produced via gas chemical reactions or physical processes) [17,24,25]. Gasses defined as primary air pollutants are potential precursors for secondary air pollutants [24,25]. Primary air pollutants include particulate matter (PM), ammonia (NH_3_), black carbon (BC), carbon monoxide (CO), methane (CH_4_), non-methane VOC (NMVOC), nitrogen oxides (NOx), or sulfur dioxide (SO_2_) [26]. Secondary air pollutants include PM, ozone (O_3_), nitrogen dioxide (NO_2_), and oxidized VOC [27] (Figure 2).

Air pollution can cause various disorders, including acute respiratory infection, chronic obstructive pulmonary disease, stroke, lung cancer, or ischemic heart disease [17,28].

### 3.2. Clinical Evidence of Air Pollution Associated with Cardiac Arrhythmias

Recent evidence shows that exposition to air pollutants is associated with the development of cardiovascular diseases [29]. In a recent article published in The Lancet, it has been reported that air pollution is responsible for about 19% of total deaths related to cardiovascular disease (CVD) [30]. CVD is described as one of the major AF risk factors [1,31]. In 2019, Kwon et al., demonstrated, that in a nationwide cohort from the Korean general population, short-term exposure to 10-µg/m^3^ increase in ambient air pollutant PM_2.5_ (particulate matter ≤ 2.5 μm in aerodynamic diameter) was associated with a significant 4.5% increase of emergency visits for AF [32]. These conclusions were consistent with the observations made by Hsiu Hao Lee et al., in 2019, reporting that, in a cohort of 670 patients from Taiwan, short-term exposure to air pollutant PM_2.5_ was associated with about 20% increase in hospitalization for AF in the 2-first days following exposure [10]. More recently, in an article published in 2020, Adjani A. Peralta and collaborators reported that exposure to PM_2.5_ was associated with a 39% increase in hospitalization for ventricular arrhythmias (VA) in a cohort of 176 VA patients from Boston, in the United States of America [33]. In 2021, in a meta-analysis evaluating 18 studies, Chao Yue and colleagues discovered that air pollutant exposure is associated with an increased prevalence of AF in the general population [34]. In Canada, a retrospective study by Saeha Shin and collaborators revealed that air pollution was associated with an increased incidence of stroke and AF [35] (Table 1).

Globally, air pollution is a major concern associated with serious CVD events, including cardiac arrhythmias and AF, leading to decreased quality of life. Hence, novel socio-cultural strategies, new lifestyle habits, and innovative therapeutic approaches are required to decrease and prevent air pollution and the associated respiratory and cardiovascular disorders.

### 3.3. Specific Situation of Firefighters

When addressing the impact of air pollution on the incidence of cardiovascular disease and cardiac arrhythmia, a firefighter is one of the specific professions that come to mind, because the protagonists are frequently exposed to important concentrations of inhalable hazardous components, including aldehydes, benzene, CO, dichlorofluoromethane, hydrogen chloride, hydrogen cyanide, SO_2_, and PM [41,42]. It has been shown that during fire suppression activities, firefighters are more likely to develop CVD abnormalities, including thrombus formation, associated with acute myocardial infarction [41]. Firefighters constitute a unique population affected by air pollutants, as they are subjected to both personal and occupational exposure, which represents an enhanced risk to their cardiovascular health [43]. It has been reported that coronary heart disease is responsible for 39% of on-duty deaths among firefighters in the USA [44]. In 2021, Steven M. Moffatt and collaborators reported that sudden cardiac events are the major risk factor for duty-associated death (~50%) in the firefighter population [45,46]. In a cohort of 10860 active firefighters from the USA, the prevalence of AF was significantly increased with the number of fires fought per year, from 2% (<5 fires per year) to 4.5% (>31 fires per year) [43].

Although firefighters are a professional group significantly affected by air pollution, it is important to study and recognize the potential impact of occupational exposure on CVD and cardiac health in other activities, including people working in agriculture, gas refineries, or ores [17,47]. In this context, various studies suggest an important deleterious role of other environmental pollutants, including pesticides and herbicides on human health [48]. The following sections will discuss evidence of the association between pesticide exposure and the development of CVD and cardiac arrhythmias.

## 4. Relation between Pesticide Exposure and Cardiac Rhythm Disorders

**Figure 3:**
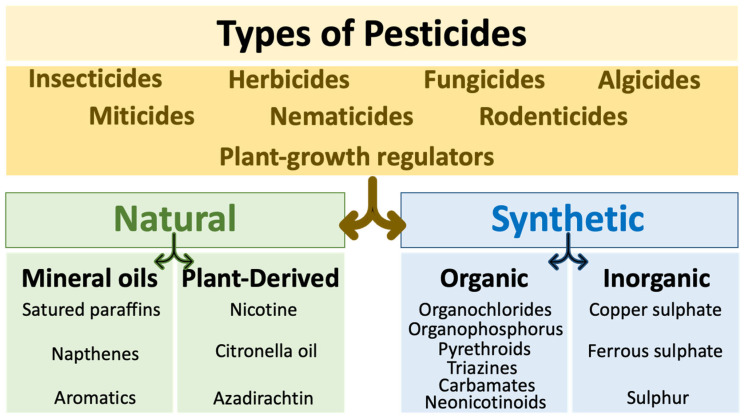
Categories of Pesticides. Pesticides, including insecticides and herbicides, can be classified as natural and synthetic. Natural pesticides can be sub-categorized according to their origin as mineral oils or plant-based pesticides. Synthetic pesticides include organic and inorganic pesticides.

The negative impact of toxic substances, including pesticides, on the environment and human and mammalian health is a major concern worldwide [48,49,50]. Mounting evidence suggests a significant implication of pesticides in the development of human disorders, including CVD [48,50]. Pesticides include various categories: insecticides, fungicides, herbicides, plant growth regulators, algicides, miticides, nematicides, and rodenticides [51,52] that can be divided into natural (mineral oils and plant-derived) and synthetic (organic and inorganic) compounds [53,54]. In terms of chemical structure, the most commonly used classes of organic pesticides include organochlorides, organophosphorus, pyrethroids, triazines, carbamates, or neonicotinoids [53,55] (Figure 3).

In this section, we will focus on the reported impact of organochlorides and organophosphorus in the development of cardiac arrhythmias and AF.

### 4.1. Organophosphorus Exposure and Cardiac Rhythm Alterations

#### 4.1.1. Clinical Reports of Organophosphorus Exposure Associated with Cardiac Arrhythmias

Organophosphates, also called organophosphorus, are esters of phosphoric acid [56]. Organophosphorus poisoning is a major clinical and public health problem worldwide, concerning developed, developing, and underdeveloped countries [57]. Various clinical reports support that acute and prolonged exposure to organophosphorus is associated with the occurrence of cardiac arrhythmias, including AF [58,59,60,61,62]. In addition to cardiac rhythm abnormalities, the manifestations of organophosphorus intoxication can also include central nervous system perturbation, acute myocardial injury, heart failure, acute renal damage, hepatic dysfunction, and respiratory disorder [63,64]. In a case report published in 2017, Dr M. Maheswari and Dr S. Chaudhary described that a patient accidentally poisoned with organophosphorus was admitted to the emergency room with acute-onset AF. It has been shown that detoxification of organophosphorus compound was accompanied by sinus rhythm recovery [58]. In a retrospective study analyzing clinical data from 98 patients admitted from 2013 to 2017 for acute exposure to organophosphorus pesticide, poisoning was associated with a significantly higher incidence of cardiac arrhythmia and heart failure compared to the control group [63]. Detoxification included gastrolavage (30 °C; 10–30 L), intravenous pralidoxime, and atropine administration (0.5 to 3 mg every 0.5 to 2 h), depending on the severity of intoxication [63].

**Figure 4:**
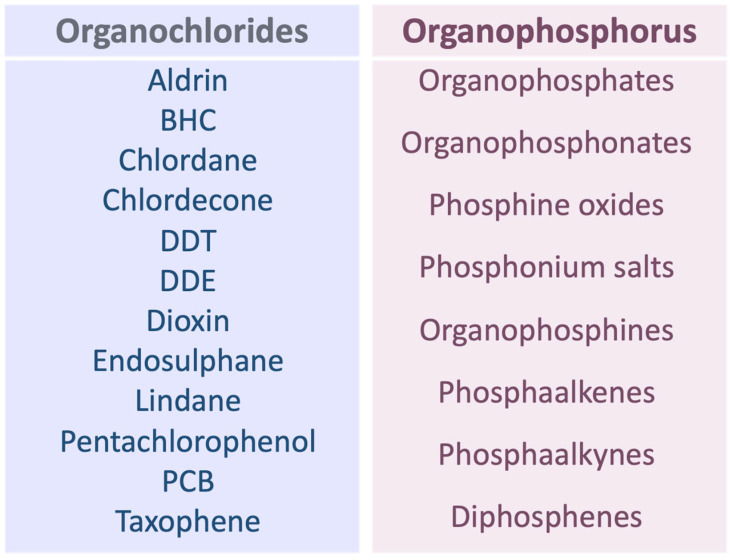
A list of the most popular Organochlorides and Organophosphorus. Organochlorides, including DDT, DDE, PCBs, and organophosphorus, including organophosphonates such as glyphosate, are among the most commonly used and commercialized pesticides worldwide. BHC: benzene hexachloride; DDT: dichlorodiphenyltrichloroethane; DDE: dichloro diphenyl dichloroethane; PCB: polychlorinated biphenyls.

Organophosphorus compounds are diverse and according to their chemical structure, they can be sub-classified as organophosphates, organophosphonates, phosphine oxides, phosphonium salts, organophosphines, phosphaalkenes, phosphaalkynes, or diphosphenes [65]. A particular organophosphonate called glyphosate is a very popular and commercially available herbicide reported to have serious carcinogenic effects [66,67,68,69] (Figure 4). In the following section, we discuss the association between glyphosate exposure and the incidence of cardiac rhythm disorders.

#### 4.1.2. Organophosphonate Exposure: Particular Case of Glyphosate Poisoning and Cardiac Rhythm Disorders

Glyphosate is a phosphonate glycine. Its molecular weight is 169.073 g/mol. The chemical structure of glyphosate includes monobasic (carboxylic) and dibasic (phosphonic) acidic sites and an amino acid glycine [69] (Figure 4). The primary target of glyphosate is the shikimate pathway, which produces the aromatic amino acids phenylalanine, tyrosine, and tryptophan in plants and microorganisms [70]. When glyphosate started to be commercialized, due to its specific effects on vegetables, its toxicity on mammals, including animals and humans was minimized or unsuspected [66]. However, numerous case studies demonstrating adverse consequences of glyphosate exposure in patients started to emerge [66,71]. Studies have shown that excessive exposure and high plasma concentrations of glyphosate can lead to severe cardiac, liver, and kidney injuries [11,72,73]. In the heart, studies have shown that exposure to glyphosate is a contributing factor in a variety of electrophysiological depolarization and repolarization conduction problems, such as a prolonged QTc, intraventricular block, and atrioventricular (AV) conduction delay [11,74]. These alterations contribute to the development of life-threatening arrhythmias, including tachyarrhythmia, atrial fibrillation, or ventricular fibrillation [75,76].

Roundup is a widely commercialized glyphosate-based herbicide [68]. Roundup residues are often detected in tap water, food, or groundwater [77]. Hence, the impact of this compound on human health is a major concern in countries where it is or has been extensively used [67]. In a case report published in 2020 by Dr Brunetti and collaborators, a patient who used 50% concentrate Roundup without gloves for weeks was hospitalized and ECG showed significantly prolonged QTc, prolonged PR interval, and first-degree AV block [11]. Although extensive data are available about the association between Roundup exposure and the development of cancer, reproductive system, respiratory system, and cardiac function, more investigations are required to characterize its impact on AF incidence.

### 4.2. Organochlorus Exposure and Cardiac Arrhythmias

#### 4.2.1. Association between Organochlorus Exposure and Cardiac Arrhythmias

Organochlorine pesticides are synthetic compounds used worldwide, in agricultural and industrial applications [78]. It was reported that 40% of all pesticides commonly used, belong to the organochlorine group [78]. The most frequently used organochlorine pesticides include molecules such as dichlorodiphenyltrichloroethane (DDT), dichloro diphenyl dichloroethane (DDE), chlordane, lindane, aldrin, benzene hexachloride (BHC), chlordecone, dioxin, endosulphane, pentachlorophenol, polychlorinated biphenyls (PCBs), taxophene (Campheclor) [78,79] (Figure 4). They are classified by the World Health Organization (WHO), as hazardous, with potential toxic effects on human health [80]. Organochlorine pesticide toxicity is characterized by their high persistence, due to low solubility in aqueous environments and high solubility in lipid areas [78]. Persistent organochlorine pollutants (POPs) are a major concern because the general population is quasi-constantly exposed to low, moderate, or high doses via alimentation, through water consumption, vegetables, animal meat, fish fats, or milk products [81,82,83].

In a mice model of atherosclerosis, PCB administration was associated with increased angiotensin II-induced aortic aneurysm and atherosclerotic lesions [84]. Also in mice, dioxin administration was associated with increased systemic hypertension and left ventricular hypertrophy [85]. In mice, PCB administration was associated with cardiac hypertrophy and abnormal blood pressure [86]. A Clinical report suggested that lindane ingestion (accidental or intentional) was associated with the occurrence of atrial fibrillation and flutter [87]. A study of the frog atrium suggests that lindane-associated rhythm disorder may reside in the fact that lindane increases rapid delayed outward K^+^ currents which provokes action potential repolarization in the atria [88].

More basic research and fundamental investigations are required to better characterize and understand the association between organochlorine exposure and the incidence of cardiac arrhythmias. In the next section, we discuss the evidence of the role of chlordecone, a yet forbidden organochlorine, on the occurrence of cardiac arrhythmias. This compound offers an interesting perspective of analysis, because it has been intensively used, and then abolished. Hence, we can retrospectively and prospectively evaluate the impact of its exposure and the consequences of its effects on human health even years after its utilization.

#### 4.2.2. Focus on the Chlordecone Cardiotoxicity

Chlordecone (CLD), also known as Kepone, is an organochlorine pesticide that was intensively used in various industrialized countries from 1972 to 1993, particularly in the United States, South America, and the Caribbean, to repel an insect known as the black banana weevil [89,90,91]. CLD is classified as a persistent organic pollutant (POP) identified as carcinogenic in the Stockholm Convention of 2009 [92,93,94]. The utilization and commercialization of CLD—except for research purposes—is currently forbidden worldwide [94]. Although this molecule is no longer used for two decades, significant concentrations persist in the soils and groundwaters of countries where CLD has been spread, making it one of the main pollutants found in table water and frequently found in rivers [95,96]. The latent presence of CLD represents a permanent danger for the exposed populations [95,96,97]. CLD exposure has been described to be associated with an increased incidence of various disorders, including breast cancer, prostate cancer, neurodegenerative and endocrine diseases, or fertility/fetal abnormalities [91,95,96,97,98,99,100,101].

Little is known about the role of CLD in the development and/or aggravation of cardiac diseases. The lack of information does not reflect a lack of effect, but a poorly studied spectrum of the poison’s effect on human health.

**Figure 5:**
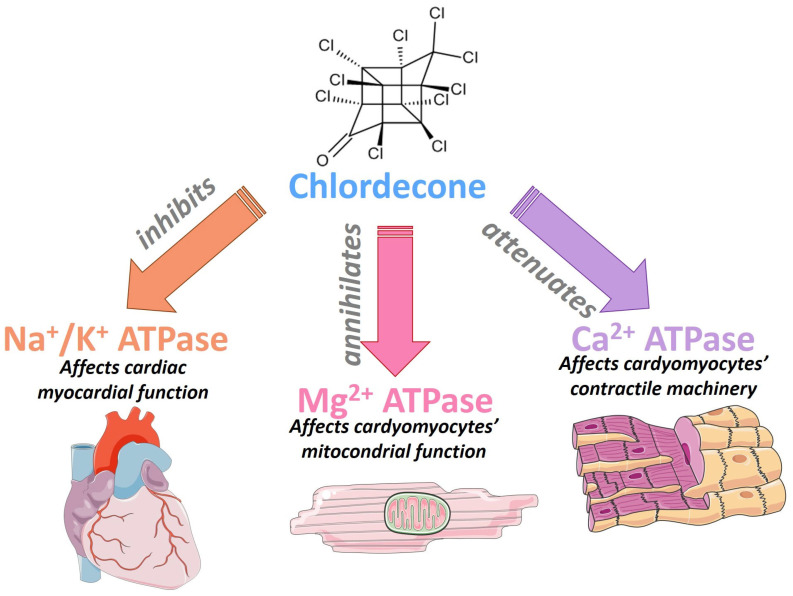
Reported effects of chlordecone on the heart. Chlordecone poisoning has been associated with dysfunction of the myocardium, cardiomyocyte mitochondrial, and calcium-dependent contractility. ATPase: Adenosine Triphosphatase; Ca^2+^: Calcium; Cl: Chlordecone; Mg^2+^: Magnesium; Na^+^: Sodium; O: oxygen.

Recent data suggest that CLD can perturbate the activity of the Na^+^/K^+^ ATPase pump in the myocardium [101,102], disturb the interaction of catecholamines with cardiac cells [99], annihilate Mg^2+^/ATPase at the cardiomyocyte mitochondrial level [101,102,103], and inhibit calcium (Ca^2+^) machinery via attenuation of Ca^2+^/ATPase and decreased sarcoplasmic reticulum calcium uptake [104]. These enzymes play a crucial role in normal myocardial physiology and homeostasis of cardiac activity [105]. Moreover, it has been shown that the deregulation of Na^+^, K^+^, Ca^2+^, or/and Mg^2+^ is responsible for the development and aggravation of cardiac diseases including AF [106,107,108] (Figure 5).

## 5. Proposed Mechanisms Underlying the Association between Pesticide Poisoning and the Occurrence of Cardiac Arrhythmias and AF

### 5.1. Generalities

**Figure 6:**
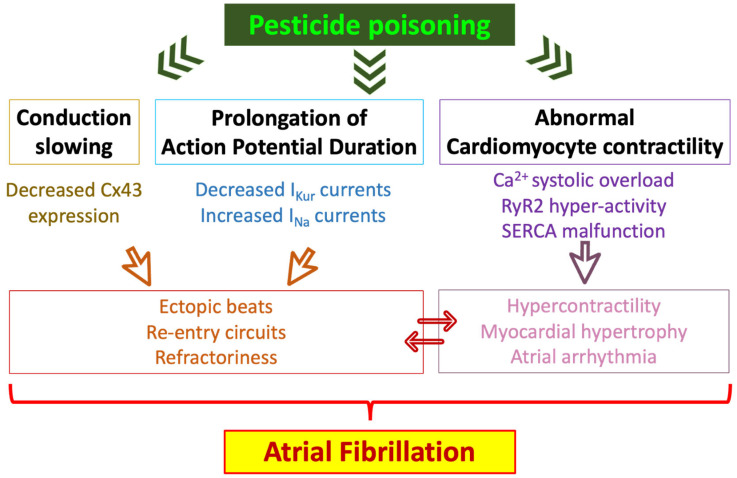
Possible mechanisms associating pesticide exposure to the development of atrial fibrillation. Ca^2+^: calcium; Cx43: connexin 43; I_Kur_: ultra-rapid delayed rectifier outward potassium current; I_Na_: inward sodium current; RyR2: ryanodine receptor; SERCA: sarco/endoplasmic reticulum Ca^2+^-ATPase.

The mechanisms involved in the pathophysiology of AF have been described at the molecular, cellular, and tissular levels. Conditions affecting the atria can lead to arrhythmia and AF when cardiac remodeling involves malfunction of ion channels implicated in the elaboration of the action potential, conduction anomalies, occurrence of electrical re-entry, or development of atrial fibrosis [109]. Pesticides and pollutants are suspected to provoke systemic or/and cardiac inflammation, oxidative stress, or cardiac structural remodeling [15,110]. Studies recently assessed the effects of air pollution on the induction of AF [110,111]. Reports suggest that air pollutants including PM, CO, H_2_S, SO_2_, O_3_, or NO_2_ may provoke cardiac arrhythmias or AF by (i) perturbating electrical conduction via attenuation of connexin-43 function, (ii) reduction of I_Kur_ currents, (iii) increase of I_Na_ currents, (v) promotion of Ca^2+^ cytosolic overload, (vi) enhancement of RyR activity, (vii) reduction of SERCA function, (viii) increase of action potential duration and (vii) by prolongation of QT intervals [110,111] (Figure 6).

### 5.2. Proposed Concept of Pollutant-Induced Cardiac Inflammation and AF

Investigations focusing on the impact of pollutants on the development of cardiac arrhythmias and AF are needed. Fundamental data and experimental models of air pollution, organochloride, and organophosphate exposure will help to better understand the impact of such poisoning on cardiac health. Although speculative but supported by the current knowledge available, we propose here a possible cascade of molecular and cellular events that may occur following exposure to environmental poisons, which may lead to cardiac arrhythmias and AF.

When an individual is exposed to inhaled or consumed environmental pollutants, pathogen-associated molecular patterns (PAMPs), or damage-associated molecular patterns (DAMPs) production increases in the organism [109]. PAMPs and DAMPs are recognized by pattern-recognition receptors, which are expressed on the surface membrane of cardiac cells, including macrophages, neutrophils, endothelial cells, cardiomyocytes, or fibroblasts [112]. The activity of pattern-recognition receptors promotes the activation of various pro-inflammatory signals involving a variety of intracellular inflammasome complexes, including the NACHT, LRR, and PYD domains-containing protein-3 (NLRP3) [113]. The NLRP3 inflammasome is implicated in the development and progression of multiple cardiovascular maladies such as systemic hypertension, myocardial infarction, or cardiac arrhythmias [114]. The assembled and activated NLRP3 inflammasome promotes the maturation of the inactive isoforms pro-interleukin-(IL)-1β and pro-IL-18 into active IL-1β and IL-18 [109,113,114,115]. Moreover, NLRP3-induced gasdermin-D (GSDMD) cleavage into N-terminus GSDMD (GSDMD-Nt) generates the formation of pores through the cellular membrane allowing the products of the inflammasome activity, including IL-1β and IL-18 to be excreted and play further autocrine, paracrine, and endocrine pro-inflammatory signaling [116]. Furthermore, studies have shown that the blood level of circulating N-terminal pro-brain natriuretic peptide (NT-proBNP) is significantly increased in patients following short-term and long-term air pollution exposure [117,118]. An abnormally elevated level of NT-proBNP is well-accepted as a predictor of cardiovascular events and cardiac arrhythmias, including AF [119,120]. Although few data are available, studies support that a trifactorial relation may exist between air pollution, increased NT-proBNP levels, and the incidence of AF [117].

**Figure 7:**
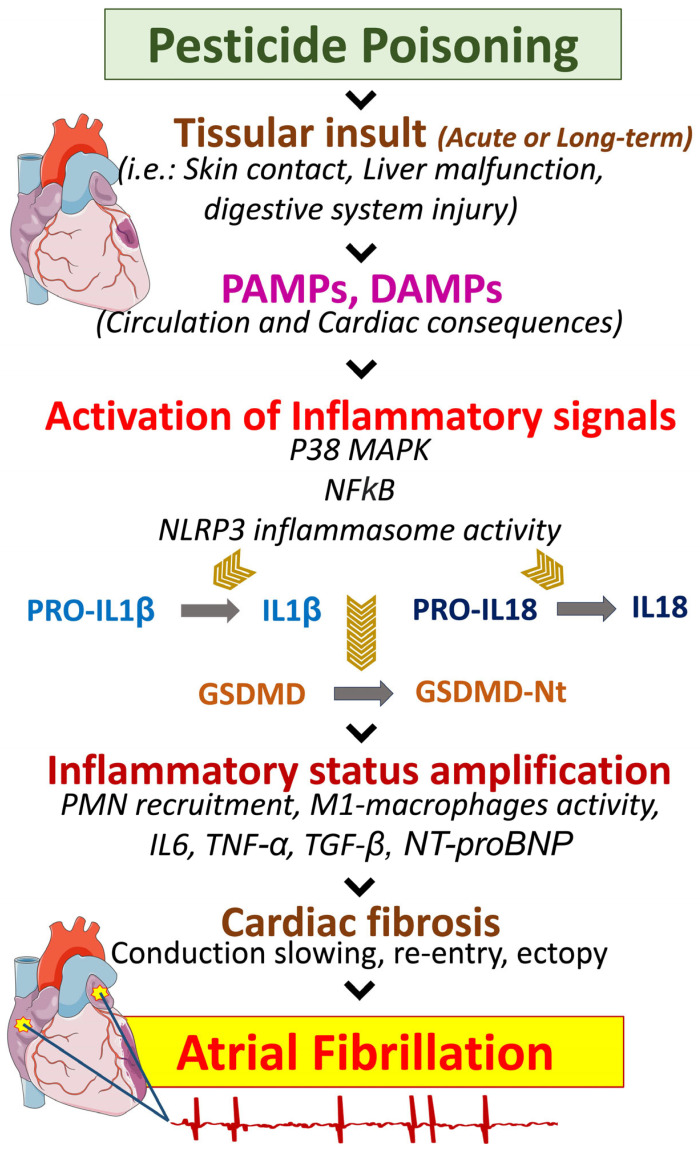
A potential implication of the inflammatory response to pesticide poisoning, leading to atrial fibrillation. Pesticide poisoning provokes tissular injury following primary contact with skin, eyes, or digestive system. The toxic interaction leads to local injury and reaction provoking tissular damage signals impaired by DAMPs and PAMPs, which lead to the activation and the excretion of inflammatory signals that can affect other systems, via the blood circulation. Reported affected systems include, among others, the brain, the liver, the kidney, and the heart. Circulating pesticide metabolites and inflammatory signals may promote the amplification of cardiac inflammatory status, which, if unresolved can lead to chronic inflammation and cardiac fibrosis. Fibrosis is associated with abnormal conduction and atrial refractoriness, which contributes to increasing the risk of cardiac arrhythmias and atrial fibrillation. DAMPs: damage-associated molecular patterns; GSDMD: gasdermin-D; IL: interleukin; NFkB: nuclear factor kappa B; NLRP3: NACHT, LRR, and PYD domains-containing protein-3; Nt: N-terminal; P38 MAPK: p38 mitogen-activated protein kinases are a class of mitogen-activated protein kinase; PAMPs: pathogen-associated molecular patterns; PMN: polymorphonuclear neutrophils; TGF-b: transforming growth factor beta; TNF-a: tumor necrosis factor-alpha.

If untreated and unresolved, the inflammatory status may lead to cardiomyocytes-, fibroblasts- and pro-inflammatory-(M1)-macrophage-induced production of inflammation-related compounds such as IL-6, TNFα, or NF-kB [121,122,123,124]. In the heart, such inflammatory signaling coupled with evident poisoning-induced oxidative stress has been demonstrated to promote abnormal calcium-Ca^2+^-handling, RyR2 dysfunction, delayed or shortened repolarization, triggered action potential, shortened effective refractory periods, and atrial fibrosis [15,109,125,126]. The chronicity of this deleterious cascade can provoke the formation of cardiac fibrosis, myocardial hypertrophy, and gap-junction lateralization provoking atrial electrical abnormalities such as ectopic activity and re-entry, leading to increased susceptibility to AF [15,109,125,127] (Figure 7).

## 6. Perspectives and Management

Studies have evaluated acute versus long-term pesticide exposure to pesticides and the incidence of cardiovascular events and the incidence of AF [38,128]. However, very few data are available about the acute and long-term cardiac remodeling induced by pesticides leading to AF [128]. In other words, although we have a comprehensive idea of acute exposure associated with acutely induced AF, the currently available knowledge is limited about whether acute exposure can lead to long-term damages that may increase the risk of AF.

Our current review article reports and discusses studies that have reported the incidence of AF following pollutant exposure, but an interesting perspective would be to perform an additional systematic review of acute and long-term pollutant damages related to AF. In such type of review article, it would be important to consider pollutant concentration (acute and long-term) and their consequences in inducing acute and long-term damages responsible for AF episodes.

**Figure 8:**
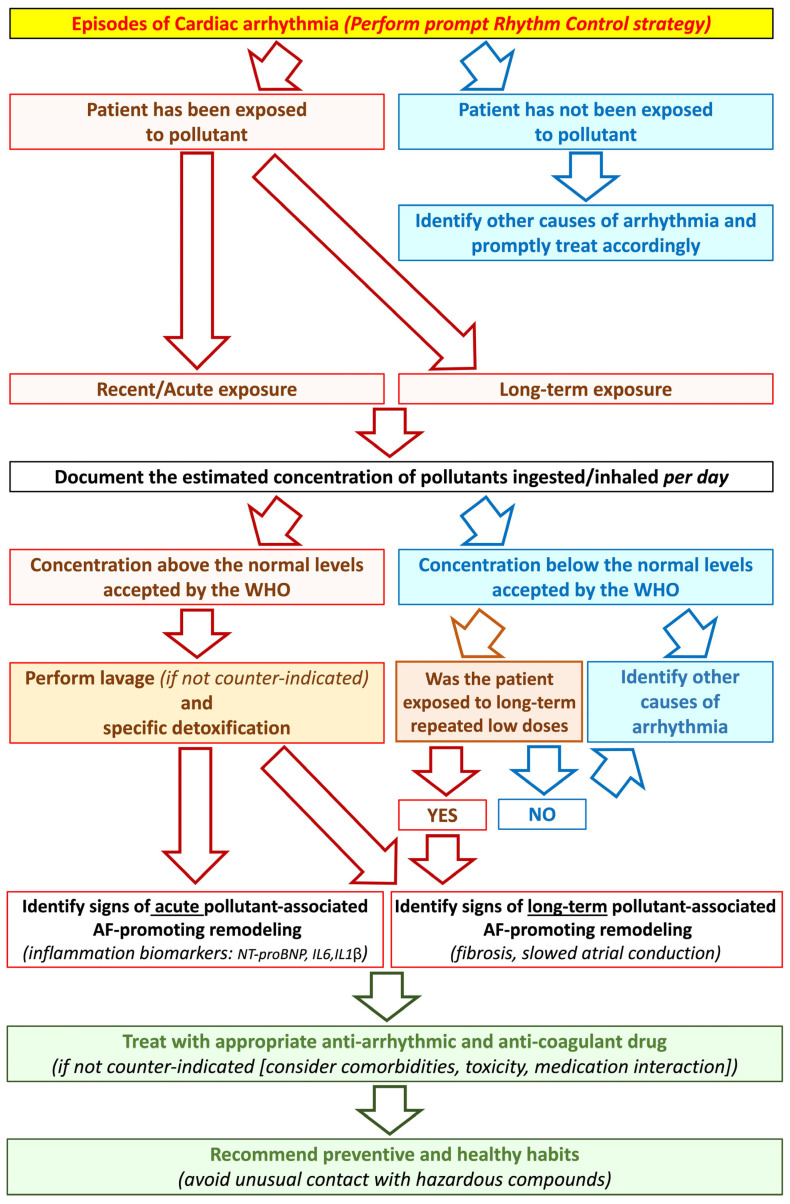
A proposed algorithm for AF-patient management following pollutant poisoning. AF: atrial fibrillation; NT-proBNP: N-terminal pro-brain natriuretic peptide; IL: interleukin; WHO: World Health Organization.

Here we propose an algorithm that may help to diagnose and manage AF following acute or long-term pollution exposure (Figure 8). When a patient arrives at the hospitalization room with cardiac arrhythmia, including AF, the care provider must identify whether the patient was recently exposed to abnormally elevated levels of pollutants [38,127,128]. The history of pollution exposure should also be questioned when interrogating the patient about his/her potential long-term exposure to pollutants (occupational [agriculture, firefighter, garbage collector]; habitat [polluted cities, near gas emission factories, near highways]) [22,43,47]. Immediate decontamination of the patient should be performed, often via gastric lavage, to evacuate pollutants from the system [129]. Prompt rhythm control strategies should be applied to promote sinus rhythm recovery. Medications should be used with caution to avoid non-recommended chemical interactions. If not counter-indicated, anticoagulation can be used to avoid clot formation and prevent stroke [130].

## 7. Conclusions

The impact of pollution on cardiac health is a major concern worldwide, due to the important and irreversible environmental and behavioral changes faced by humans and all living beings. Clinical and fundamental research have demonstrated that the toxicity of commonly used pesticides, or herbicides is related to the development of cardiac arrhythmias including AF. Although the mechanisms of pesticide-induced AF remain unclear, more investigation will help to better understand how to prevent and cure pollution-associated arrhythmogenicity.

## 8. Future Directions and Call for Action

Mounting evidence suggests that air pollutants and some pesticides/herbicides/insecticides including compounds of the organophosphorus and organochloride categories may lead to acute or chronic cardiac rhythm disorder following ingestion or inhalation of elevated concentrations. Populations concerned by primary services activities, or occupational intoxication due to frequent utilization and close contact with pesticides (home gardening, agriculture) must be considered and observed thoroughly to prevent the risk of cardiac disorder and AF [125,126]. Although few data are available, this narrative review article is a “call for action” to assess the urgent need for more fundamental and clinical research evaluating the association between environmental pollutants and the risk of cardiac toxicity, arrhythmias, and AF. Such studies will help to improve the diagnosis and management of AF while contributing to ameliorating the guidelines, policies, and recommendations in terms of pesticide utilization.

## Figures and Tables

**Table 1:** Clinical studies highlighting the association between air pollution and cardiac arrhythmias. AF: atrial fibrillation; CO: carbon monoxide; NO_2_: nitrogen dioxide; O_3_: ozone; PM: particulate matter; SO_2_: Sulfur dioxide; VA: ventricular arrhythmias.

Pollutant	Patient Population				Induced Arrhythmia (Incidence/Prevalence)	Reference
	Total	Age (Years)	Sex	Country		
PM_2.5_	124,010 patients	48.5 ± 12.5	Male: 48.8% Female: 51.2%	South Korea	AF 95% CI = 1.02–1.09	Kwon OK et al., 2019 [32]
PM_2.5_	670 patients	70.5 ± 14	Male: 51% Female: 49%	Taiwan	AF 95% CI = 1.03–1.44	Lee HH et al., 2019 [10]
PM_2.5_	176 patients	60 ± 20	Male: 77% Female: 23%	USA	VA 95% CI = 1.15–1.90	Peralta A et al., 2020 [33]
PM_2.5_	Meta-analysis (18 studies)			USA Canada Europe Asia	AF 95% CI = 1.01–1.10	Yue C et al., 2021 [34]
PM_2.5_; NO_2_ O_3_; Ox	5,071,956 patients	53.2 ± 12.9	Male: 48% Female: 52%	Canada	AF 95% CI = 1.01–1.04	Shin S et al., 2019 [35]
PM_2.5_; PM_10_ SO_2_; NO_2_ O_3_; CO	176 patients	58 ± 32	Male: 70% Female: 30%	USA	AF 95% CI = 1.08–1.47	Link MS et al., 2013 [36]
PM_2.5_; SO_2_; NO_2_ O_3_	Meta-analysis (18 studies)			USA Europe Asia	AF 95% CI = 1.02–1.06	Chen M et al., 2021 [37]
PM_2.5_	125 patients	77.6 ± 7.8	Male: 61.5% Female: 38.5%	Sweden	AF 95% CI = 1.01–1.10	Dahlquist M et al., 2022 [38]
NO_2_	369 patients	66.3 ± 15.9	Male: 46.9% Female: 53.1%	Iran	AF 95% CI = 1.02–1.55	Saifipour A et al., 2019 [39]
PM_2.5_; PM_10_	145 patients	70.5 ± 6.5	Male: 75.2% Female: 24.8%	Italia	AF 95% CI = 1.34–4.28	Gallo E et al., 2020 [40]

## Data Availability

Not applicable.
